# Unveiling the Bio-Interface via Spectroscopic and Computational Studies of (Propyl-3-ol/butyl-4-ol)triphenyltin(IV) Compound Binding to Human Serum Transferrin

**DOI:** 10.3390/ma19030457

**Published:** 2026-01-23

**Authors:** Žiko Milanović, Emina Mrkalić, Jovan Kulić, Goran N. Kaluđerović

**Affiliations:** 1 Institute for Information Technologies, University of Kragujevac, Liceja Kneževine Srbije 1A, 34000 Kragujevac, Serbia; emina.mrkalic@pmf.kg.ac.rs; 2 Faculty of Medicine Foča, University of East Sarajevo, 73300 Foča, Bosnia and Herzegovina; jovan.kulic@ues.rs.ba; 3 Department of Engineering and Natural Sciences, University of Applied Sciences Merseburg, Eberhard-Leibnitz-Straße 2, 06217 Merseburg, Germany

**Keywords:** organotin(IV) compounds, triphenyltin(IV) compounds, transferrin binding, protein fluorescence quenching, ONIOM QM/MM

## Abstract

Two structurally tunable (propyl-3-ol)triphenyltin(IV) (**Ph_3_SnL_1_**) and (butyl-4-ol)triphenyltin(IV) (**Ph_3_SnL_2_**) compounds were investigated at the human serum transferrin (**Tf**) molecular interface to resolve how ligand architecture and protein metallation modulate organotin(IV) biocompound stability and lobe-selective binding. Steady-state fluorescence spectroscopy revealed efficient quenching of native **Tf** emission (λ_ex_ = 280 nm, 296–310 K, pH 7.4) without significant spectral displacement, indicating the predominant formation of non-fluorescent ground-state complexes. Calculated bimolecular quenching constants (*K*_q_ ~10^12^ M^−1^ s^−1^) exceeded the diffusion-controlled aqueous limit, ruling out a collisional dynamic quenching mechanism and confirming static complexation as the principal origin of fluorescence suppression. Double-log binding analysis revealed moderate affinity (*K*_a_ ~10^2^–10^3^ M^−1^) and an approximately single dominant binding event per protein (*n* ≈ 0.65–0.90). Temperature-dependent van’t Hoff evaluation yielded positive Δ*H*° and Δ*S*° values, supporting a spontaneous, entropy-favored association process largely governed by hydrophobic and dispersion-type contributions, consistent with lipophilic organotin(IV) scaffold accommodation. Iron (Fe^3+^) loading of **Tf** markedly enhanced ligand engagement, especially for **Ph_3_SnL_1_**, evidencing that metallation-induced lobe closure reshapes pocket accessibility and local polarity relevant for organotin(IV) binding presentation rather than simply strengthening empirical docking scores. Molecular docking localized the most stable **Ph_3_SnL_2_** poses in the sterically confined, rigid C-lobe, while **Ph_3_SnL_1_** preferentially penetrated the more adaptive N-lobe. ONIOM QM/MM refinement of docking poses confirmed strong interfacial stabilization (Δ*E*_int_ ≈ –38 to –62 kcal mol^−1^) and clarified charge–packing interplay without invoking frontier orbital analysis. The results map multiscale structure–interaction relationships defining lobe preference and complex stability at the transferrin interface.

## 1. Introduction

Organotin(IV) compounds constitute a prominent class of organometallic materials distinguished by their pronounced structural diversity and physicochemical tunability, which underpin a wide spectrum of applications spanning catalysis, materials science, and bioinorganic chemistry [1,2,3,4]. Within this family, triphenyltin(IV) derivatives have received sustained attention owing to the modular nature of their ligand environments, versatile coordination behavior, and well-documented biological activity [5,6,7]. In biological settings, however, the functional performance of organotin(IV) species is not dictated solely by their intrinsic molecular properties but rather by a complex interplay of processes occurring at bio-interfaces [8,9]. The term “bio-interface” denotes the dynamic contact region between the compound and the protein surface, where non-covalent interactions and protein conformational adaptability govern molecular recognition and binding stability. At these interfaces, the ligand architecture, steric demand, lipophilicity, and electronic structure collectively govern the stability of compounds, their transport pathways, and modes of interaction with biomacromolecules, such as proteins and membranes [10]. Subtle structural variations may therefore translate into markedly different biological responses [11]. Elucidating these interfacial phenomena is crucial for the rational design of metal-containing materials that can maintain structural integrity and functional efficacy in complex biological matrices, as well as for establishing reliable structure–property–function relationships relevant to biomedical and bio-inspired applications.

**Figure 1:**
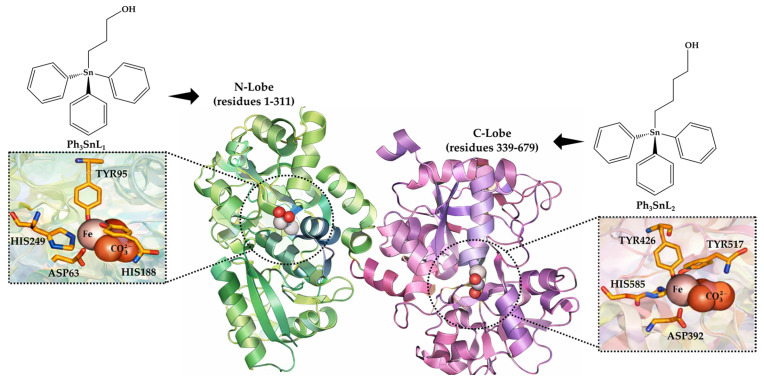
Bio-interfacial landscape of Human Serum Transferrin (**Tf**) as a lobe-discriminative platform for investigating triphenyltin(IV) compound (**Ph_3_SnL_1_** and **Ph_3_SnL_2_**) recognition and iron-cleft accessibility.

Human serum proteins constitute the first and most relevant biological barriers encountered by metal-based agents upon entry into systemic circulation [12]. Among them, human serum transferrin (**Tf**), a bilobal glycoprotein responsible for the physiological transport of Fe^3+^ ions, is of particular relevance in this context (Figure 1) [13]. Due to its well-defined metal-binding pockets, pronounced conformational flexibility, and exceptionally high affinity for Fe^3+^, **Tf** often serves as a key mediator of metal trafficking, cellular uptake, and the biodistribution of a wide range of coordination compounds [14]. Accordingly, **Tf** has been extensively investigated as a carrier and binding partner for a broad spectrum of endogenous substrates, small-molecule drugs, and metal-based complexes, with many non-covalent transferrin–ligand systems exhibiting moderate binding affinities, typically in the micromolar to millimolar range [15,16,17,18]. In such systems, ligand size, lipophilicity, and flexibility, together with the conformational state of the protein, govern binding strength and site preference through a balance of electrostatic, hydrogen-bonding, and hydrophobic contributions. Importantly, the reversible structural transitions between the *apo*- and *holo*-forms of **Tf** induce substantial changes in the polarity, topology, and accessibility of its binding clefts, thereby directly modulating the recognition and binding of exogenous metal compounds. While numerous studies focus on ligands that directly engage iron-coordination motifs or polar regions of the binding clefts, considerably fewer address how hydrophobic organometallic scaffolds interact with **Tf** in a lobe-selective manner. Collectively, these features establish **Tf** as a highly relevant and biologically meaningful model bio-interface for investigating the behavior of functional organometallic compounds under physiologically relevant conditions.

In recent years, the integration of spectroscopic and computational approaches has become indispensable for elucidating the molecular basis of protein–ligand interactions. Among these techniques, fluorescence spectroscopy offers a highly sensitive and quantitative means to assess binding affinity, quenching mechanisms, and the underlying thermodynamic driving forces [19]. In parallel, molecular docking and molecular dynamics (MD) simulations provide atomistic insight into preferred binding modes, ligand-induced conformational rearrangements, and the long-term stability of protein–ligand compounds [20]. Complementary quantum-chemical calculations further enhance mechanistic interpretation by enabling detailed analysis of electronic structure, frontier molecular orbitals, and reactivity descriptors. Together, these integrative strategies, fully aligned with contemporary trends in functional materials design, effectively bridge experimental observations and theoretical modeling, yielding a comprehensive understanding of structure–property–function relationships at biologically relevant interfaces.

Despite the extensive body of literature on the transferrin binding of metal-based and organometallic compounds, most previous studies have focused on global binding affinities or iron-coordination-driven recognition, often treating the protein as a homogeneous binding environment. Systematic investigations addressing how subtle variations in hydrophobic organotin ligand architecture influence lobe-selective recognition, interfacial stabilization mechanisms, and the differential response of apo- and holo-transferrin remain notably scarce. In particular, the combined assessment of fluorescence-derived thermodynamic parameters with multiscale computational validation of bio-interfacial stabilization has not been comprehensively explored for triphenyltin(IV) systems.

In this work, we investigate the bio-interfacial behavior of (propyl-3-ol)triphenyltin(IV) (**Ph_3_SnL_1_**) and (butyl-4-ol)triphenyltin(IV) (**Ph_3_SnL_2_**) compounds (Figure 1), using a multidisciplinary workflow that integrates steady-state fluorescence spectroscopy, thermodynamic analysis, molecular docking, and ONIOM (QM/MM) calculations. Within this framework, molecular docking is employed as a qualitative structural tool to rationalize binding modes and lobe selectivity, experimental fluorescence data provide solution-phase thermodynamic parameters, and ONIOM QM/MM calculations offer higher-level energetic validation of interfacial stabilization. **Tf** was selected as a model protein to systematically assess how subtle variations in ligand architecture influence binding affinity, fluorescence quenching pathways, and overall complex stability. Emphasis was placed on elucidating the role of Fe^3+^ ions, given the critical regulatory function of metal-induced conformational changes in **Tf** and their potential impact on ligand recognition.

The obtained results provide a comprehensive molecular-level description of the interactions between these organotin(IV) materials and **Tf**, revealing lobe-selective binding preferences, the predominance of hydrophobic driving forces, and the pronounced influence of protein metallation on ligand binding behavior. By correlating spectroscopic findings with atomistic simulations and electronic structure analysis, this study establishes clear structure–interaction–function relationships that enable improved prediction of the biological performance of structurally related organometallic systems. The presented experimental–computational framework is broadly applicable and offers a robust platform for the rational design of next-generation metal-based materials with tailored bio-interfacial properties.

## 2. Materials and Methods

### 2.1. Fluorescence Spectroscopy Measurements

Fluorescence emission spectra were recorded using an RF-6000 spectrofluorometer (Shimadzu, Kyoto, Japan) equipped with a 150 W xenon lamp and a temperature-controlled quartz cuvette (path length 1.0 cm). All measurements were performed at three different temperatures (296, 303, and 310 K) to evaluate the temperature dependence of the protein–ligand interactions. The excitation wavelength was set at 280 nm, and both excitation and emission slit widths were fixed at 5 nm. Emission spectra were collected over the wavelength range of 300–450 nm. Throughout the experiments, the concentration of **Tf** (human serum transferrin, Sigma-Aldrich, St. Louis, MO, USA, ≥95% purity) was maintained at a constant 2 µM, while increasing concentrations (0–27.2 µM) of the triphenyltin(IV) compounds **Ph_3_SnL_1_** and **Ph_3_SnL_2_** (prepared and spectroscopically characterized as described in previous studies [21,22]) were added. Fluorescence titrations were carried out by successive additions of 5 µL aliquots of the ligand stock solutions into the protein solution, yielding a final volume of 2.5 mL. All measurements were performed at physiological pH (7.4). After each addition, the solution was gently mixed and allowed to equilibrate before acquiring the spectrum.

#### 2.1.1. Correction of Inner Filter Effect

To eliminate distortions arising from the absorption of excitation light and the reabsorption of emitted fluorescence (inner filter effect), all observed fluorescence intensities were corrected using Equation (1) [23]:

(1) $$F_{cor}=F_{obs}\times 10^{\frac{A_{ex}+A_{em}}{2}}$$

where *F*_cor_ and *F*_obs_ represent the corrected and observed fluorescence intensities, and *A*_ex_ and *A*_em_ represent the absorbance of protein and ligand measured at the excitation and emission wavelengths, respectively.

#### 2.1.2. Fluorescence Quenching and Binding Analysis

The fluorescence quenching mechanism was analyzed using the Stern–Volmer equation [23]:

(2) $$\frac{F_{0}}{F}=1+K_{q}\tau_{0}\left[Q\right]=1+K_{\mathrm{SV}}\left[Q\right]$$

where $F_{0}$ and F denote the fluorescence intensities of **Tf** in the absence and presence of the quencher, respectively. *K*_SV_ is the Stern–Volmer quenching constant, *K*_q_ is the bimolecular quenching rate constant, τ_0_ represents the average fluorescence lifetime of the protein in the absence of quencher (assumed to be 10^−8^ s), and [Q] is the total concentration of the ligand.

In all calculations, the total ligand concentration was used, as the concentration of free ligand was considered negligible relative to the total added amount. Given the moderate binding affinity and excess ligand regime, deviations between free and total ligand concentration do not affect linearity or extracted parameters within experimental uncertainty. The Stern–Volmer constants were obtained from the linear plots of F_0_/F versus [Q].

The binding constant (*K_a_*) and the number of binding sites (n) were determined using the double-logarithmic equation [23]:

(3) $$\log\frac{F_{0}-F}{F}=\log K_{a}+n\log\left[Q\right]$$

#### 2.1.3. Thermodynamic Analysis

Thermodynamic parameters governing the interaction between **Tf** and the investigated triphenyltin(IV) compounds were evaluated based on fluorescence measurements performed at three different temperatures (296, 303, and 310 K) [24]. The enthalpy change (Δ*H*^0^) and entropy change (Δ*S*^0^) were derived using the van’t Hoff equation:

(4) $$\ln K_{a}=-\frac{\Delta H^{0}}{RT}+\frac{\Delta S^{0}}{R}$$

where *K_a_* is the binding constant, *R* is the universal gas constant, and *T* is the absolute temperature. The values of Δ*H*^0^ and Δ*S*^0^ were obtained from the slope and intercept of the linear plot of ln *K_a_* versus 1/T, respectively. The Gibbs free energy change (Δ*G*^0^) was subsequently calculated using the following equation:

(5) $$\Delta G^{0}=-RT\ln K_{a}=\Delta H^{0}-T\Delta S^{0}$$

### 2.2. Computational Approach

#### 2.2.1. Molecular Docking Analysis of Triphenyltin(IV) Compounds with Tf

The interaction of the triphenyltin(IV) compounds **Ph_3_SnL_1_** and **Ph_3_SnL_2_** with **Tf** was investigated using a molecular docking approach implemented in AutoDock Tools 1.5.7 coupled with AutoDock 4.2.6 [25]. This computational protocol was employed to assess the binding affinity, preferred binding sites, and interaction profiles of the investigated compounds within the transferrin structure. All docking calculations were performed following a validated and reproducible workflow commonly applied in protein–ligand interaction studies [26,27,28,29].

Before docking simulations, the molecular structures of **Ph_3_SnL_1_** and **Ph_3_SnL_2_** were fully optimized using the *Gaussian16* software package [30]. Geometry optimizations were performed within the framework of DFT employing the B3LYP–D3BJ functional. The 6-311+G(d,p) basis set was applied to all non-metal atoms, while the Sn atom was described using the LANL2DZ effective core potential and its associated basis set. This computational setup provided reliable structural and electronic descriptors for subsequent docking calculations [31,32].

The three-dimensional crystallographic structure of human serum transferrin was retrieved from the RCSB Protein Data Bank (PDB ID: 3V83) on 28 May 2025 [33]. Protein preparation was conducted using BIOVIA Discovery Studio 2021 [34], which involved the removal of crystallographic water molecules, co-crystallized ligands, and non-essential heteroatoms to avoid potential interference during the docking procedure.

Docking simulations were performed within two biologically relevant regions of **Tf** corresponding to the N-lobe (subdomain I) and C-lobe (subdomain II), both recognized as key ligand-binding domains [35]. The grid box centers were set at coordinates (10.182, −14.192, −18.695 Å) for the N-lobe and (6.142, 22.102, −41.700 Å) for the C-lobe. In both cases, a cubic grid box of 50 × 50 × 50 Å with a grid spacing of 0.375 Å was employed to fully encompass the binding cavities. To examine the influence of iron coordination on ligand binding, docking calculations were conducted under two conditions: in the presence and absence of Fe^3+^ ions within the transferrin structure. The **Ph_3_SnL_1_** and **Ph_3_SnL_2_** compounds were positioned toward the predefined binding regions to ensure consistent and biologically meaningful sampling of potential interaction sites. All docking simulations were carried out using the Lamarckian Genetic Algorithm (LGA), which enables efficient conformational sampling by treating the ligand as flexible while maintaining protein rigidity [36]. The algorithm parameters included a population size of 150 individuals, a maximum of 2,500,000 energy evaluations, and 27,000 generations per docking run. A mutation rate of 0.02 and a crossover rate of 0.8 were applied in accordance with established protocols. To ensure adequate exploration of binding modes and statistical robustness, 100 independent docking runs were performed for each complex.

#### 2.2.2. ONIOM (QM/MM) Computational Protocol for Protein–Ligand Interaction Energies

QM/MM calculations were performed to investigate the interactions between the triphenyltin(IV) compounds **Ph_3_SnL_1_** and **Ph_3_SnL_2_** and **Tf** using the *Our Own N-layered Integrated Molecular Orbital and Molecular Mechanics* (ONIOM) methodology, as implemented in *Gaussian16* [30,37]. A two-layer ONIOM approach was employed, in which the transferrin protein was treated as the low-level (MM) layer, while the ligand and key amino acid residues involved in binding were described at the high-level (QM). The QM region comprised the **Ph_3_SnL_1_** and **Ph_3_SnL_2_** compounds, together with the surrounding residues that directly participate in coordination, hydrogen bonding, π–π stacking, and electrostatic interactions. This region was optimized using the theoretical model described above. The remaining part of the protein was assigned to the MM layer and described using the UFF force field, as implemented in *Gaussian16*. Link atoms (hydrogen atoms) were introduced at the QM/MM boundary to cap truncated covalent bonds between the QM and MM regions. Initial geometries of the protein–ligand complexes were obtained from the lowest-energy binding poses determined by molecular docking simulations. Geometry optimizations were carried out without symmetry constraints using standard ONIOM optimization protocols. Convergence criteria for energy and forces were set to Gaussian default tight thresholds, ensuring reliable optimization of the interfacial region.

The total ONIOM energy ($E_{\mathrm{ONIOM}}$) was calculated according to the standard subtractive ONIOM scheme:

(6) $$E_{ONIOM}=E_{\mathrm{mod}el}^{high}+E_{real}^{low}+E_{\mathrm{mod}el}^{low}$$

where $E_{\mathrm{model}}^{\mathrm{high}}$ represents the energy of the QM region calculated at the high (QM) level, $E_{\mathrm{model}}^{\mathrm{low}}$ corresponds to the energy of the same region calculated at the MM level, and $E_{\mathrm{real}}^{\mathrm{low}}$ denotes the energy of the entire system calculated at the MM level.

The interaction energy (Δ*E*_int_) between transferrin and the investigated compounds was evaluated using the following expression:

(7) $$\Delta E_{\mathrm{int}}=\Delta E_{Tf-complex}-\Delta E_{Tf}-\Delta E_{complex}$$

where Δ*E*_Tf-complex_, Δ*E*_Tf_, and Δ*E*_complex_ correspond to the ONIOM energies of the optimized transferrin–complex system, the isolated transferrin, and the isolated **Ph_3_SnL_1_** and **Ph_3_SnL_2_** compounds, respectively.

## 3. Results and Discussion

### 3.1. Fluorescence Measurements

#### 3.1.1. Fluorescence Quenching

**Figure 2:**
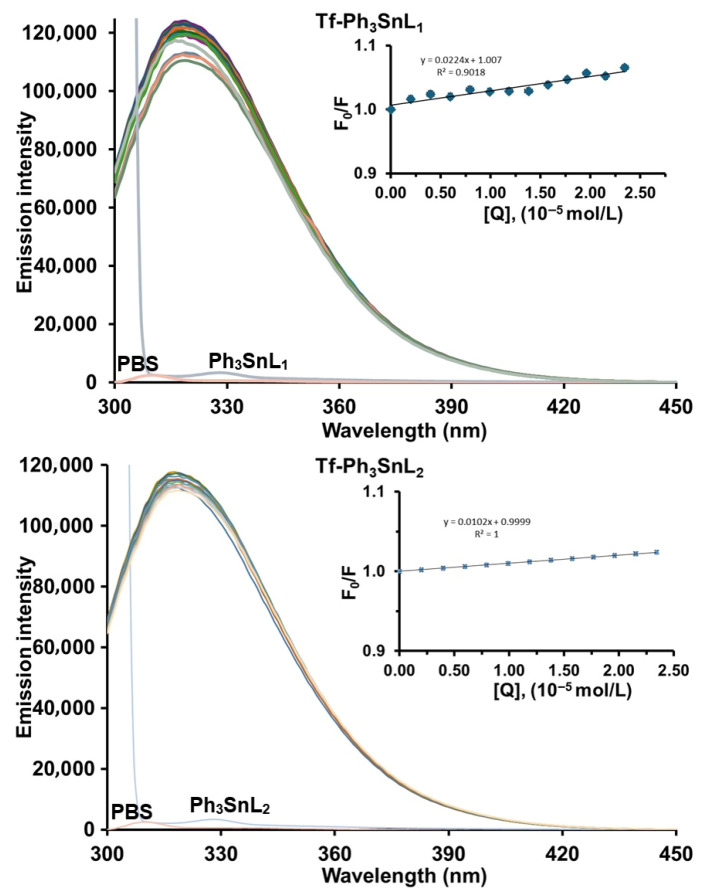
Emission spectra of **Tf** in the presence of increasing concentrations of **Ph_3_SnL_1_** and **Ph_3_SnL_2_** (T = 296 K; pH 7.4; λ_ex_ = 280 nm). [**Tf**] = 2 µM; [**Ph_3_SnL_1_] = [Ph_3_SnL_2_**] = 0–27.2 µM. The inset shows the Stern–Volmer plot.

Figure 2 and Figures S1–S4 present the fluorescence emission spectra of human serum transferrin recorded in the absence and presence of increasing concentrations of **Ph_3_SnL_1_** and **Ph_3_SnL_2_** at physiological pH (7.4) upon excitation at 280 nm. All measurements were performed at three different temperatures (296, 303, and 310 K). Native **Tf** exhibits a strong intrinsic fluorescence emission with a maximum centered at approximately 320 nm, originating predominantly from its tryptophan residues. Upon gradual addition of **Ph_3_SnL_1_** and **Ph_3_SnL_2_**, a slight decrease in fluorescence intensity is observed, while the position of the maximum remains essentially unchanged.

The absence of any significant shift in the maximum emission suggests that the microenvironment of the fluorophores is not markedly perturbed and that no major conformational rearrangement of the protein occurs upon binding. The observed quenching behavior is therefore consistent with protein–ligand association without substantial alteration of the local electronic environment of the fluorophores. Instead, the fluorescence decrease is compatible with the formation of non-fluorescent **Tf–Ph_3_SnL_1_** and **Tf–Ph_3_SnL_2_** ground-state complexes, suggesting a contribution from static quenching.

To further elucidate the quenching mechanism, Stern–Volmer quenching constants (*K*_SV_) and bimolecular quenching rate constants (*K*_q_) were evaluated at different temperatures (Table 1). For the **Tf–Ph_3_SnL_1_** system, the *K*_SV_ values tend to increase with rising temperature, whereas for **Tf–Ph_3_SnL_2_**, only minor variations in *K*_SV_ are observed upon heating. Although such temperature-dependent trends provide useful insight into the quenching process, they are insufficient by themselves to unambiguously distinguish between static and dynamic quenching mechanisms.

The magnitude of the bimolecular quenching rate constants was therefore considered as an additional supporting parameter. As listed in Table 1, the calculated *K*_q_ values for both systems are on the order of 10^11^–10^12^ M^−1^ s^−1^, which exceeds by more than two orders of magnitude the maximum diffusion-controlled quenching constant in aqueous solution (~2.0 × 10^10^ M^−1^s^−1^). This observation indicates that a purely collisional (dynamic) quenching mechanism is unlikely and that ground-state complex formation makes a significant contribution to the observed fluorescence quenching.

Taken together, these findings indicate that the quenching of transferrin fluorescence by both **Ph_3_SnL_1_** and **Ph_3_SnL_2_** is dominated by a static component associated with protein–ligand complex formation, while a minor dynamic contribution cannot be fully excluded based on steady-state fluorescence data alone. The stronger quenching efficiency observed for **Ph_3_SnL_2_**, as reflected by higher *K*_SV_ values, indicates a higher binding affinity toward **Tf**, which may be attributed to enhanced hydrophobic interactions between the ligand framework and nonpolar regions of the protein. Such hydrophobic forces are known to play a dominant role in the association of organometallic compounds with serum proteins and are consistent with the absence of significant spectral shifts.

#### 3.1.2. Binding Constant and Number of Binding Sites

**Figure 3:**
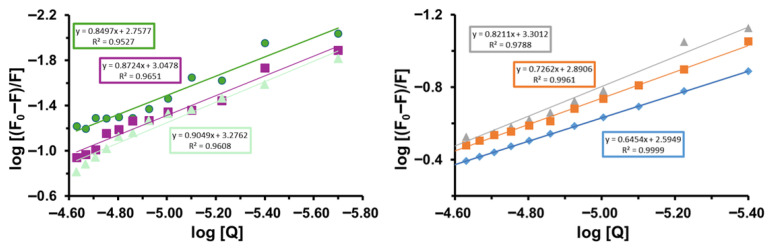
The plots of log (*F*_0_ − *F*)/*F* vs. log [Q] at different temperatures for **Ph_3_SnL_1_** (**left**) and **Ph_3_SnL_2_** (**right**).

Based on Equation (3) and the double-logarithmic plots of log[(F_0_ − F)/F] versus log[Q] obtained at three different temperatures, the binding constants (*K_a_*) and the apparent number of binding sites (*n*) for the interactions between **Tf** and **Ph_3_SnL_1_** and **Ph_3_SnL_2_** were determined. The corresponding plots are presented in Figure 3, while the extracted parameters are summarized in Table 2.

Analysis of the linear relationships with high correlation coefficients (R^2^ > 0.95) confirms the existence of specific binding sites for both triphenyltin(IV) compounds on the transferrin molecule. The values of *n* range from 0.85 to 0.90 for the **Tf–Ph_3_SnL_1_** system and from 0.65 to 0.82 for the **Tf–Ph_3_SnL_2_** system, indicating that, on average, approximately one binding site per protein molecule is involved in both interactions. Values of *n* lower than unity indicate an apparent binding stoichiometry arising from heterogeneous microenvironments and partial site accessibility rather than true sub-stoichiometric occupancy, a behavior frequently observed for small-molecule binding to large serum proteins. Such values are typical for small-molecule binding to serum proteins and suggest a relatively well-defined binding region.

The binding constants increase with temperature for both systems, with *K_a_* values ranging from 5.72 × 10^2^ to 1.89 × 10^3^ M^−1^ for **Tf–Ph_3_SnL_1_** and from 3.93 × 10^2^ to 2.00 × 10^3^ M^−1^ for **Tf–Ph_3_SnL_2_**. The comparable magnitudes of *K_a_* indicate that both complexes exhibit similar overall binding affinities toward transferrin, although **Ph_3_SnL_2_** shows a slightly stronger interaction at elevated temperatures. This temperature-dependent increase in binding strength is consistent with entropy-driven association processes.

Importantly, the magnitude of the binding constants obtained in this study falls within the range commonly reported for non-covalent interactions between small metal-based compounds and serum proteins, including transferrin and albumin, which typically exhibit binding affinities in the 10^2^–10^4^ M^−1^ range [38,39,40]. Previous studies on compound–protein and metal–protein systems have similarly reported moderate binding strengths governed primarily by hydrophobic effects and protein conformational adaptability, rather than strong site-specific metal coordination. In this context, the *K_a_* values determined for **Ph_3_SnL_1_** and **Ph_3_SnL_2_** are fully consistent with literature trends, supporting the classification of these interactions as reversible, non-covalent, and bio-interfacially mediated.

**Figure 4:**
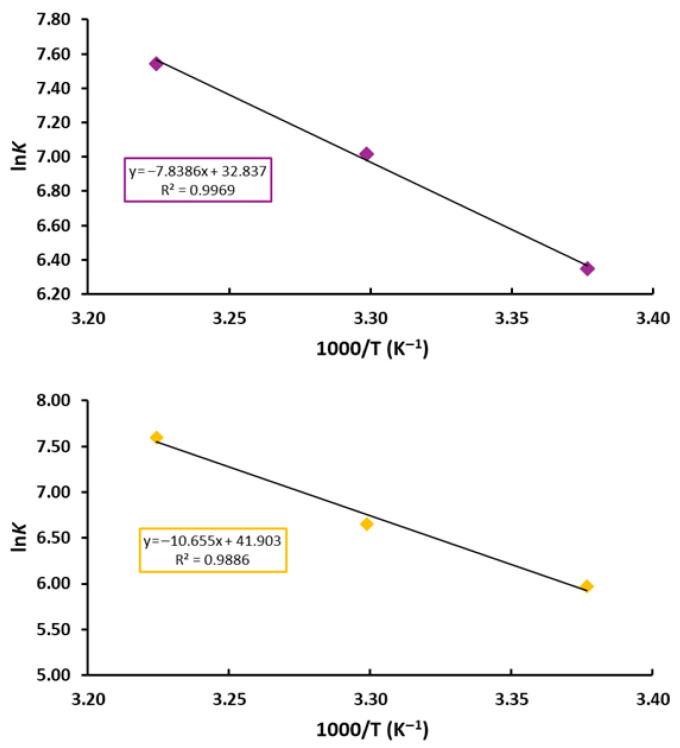
Van’t Hoff plots illustrating the temperature dependence of the binding constant (ln K) as a function of 1/T for the **Tf–Ph_3_SnL_1_** (**top**) and **Tf–Ph_3_SnL_2_** (**bottom**) systems at 296, 303, and 310 K.

To further elucidate the nature of the binding forces, thermodynamic parameters including the Δ*H*°, Δ*S*°, and Δ*G*° were evaluated. The Van’t Hoff equation was employed to derive Δ*H*° and Δ*S*° from the slope and intercept of the linear plots of ln *K_a_* versus 1/T (Figure 4). At the same time, Δ*G*° values were calculated according to the Gibbs–Helmholtz relationship.

The thermodynamic parameters summarized in Table 2 provide clear insight into the dominant interaction mechanism. The positive values of Δ*H*° (65.170 and 88.586 kJ mol^−1^) and Δ*S*° (0.273 and 0.348 JK^−1^mol^−1^) for the **Tf–Ph_3_SnL_1_** and **Tf–Ph_3_SnL_2_** systems, respectively, indicate that predominantly hydrophobic interactions, with additional dispersion and packing contributions, play a major role in stabilizing the protein–ligand complexes. Such an entropically favored binding process is commonly associated with the release of ordered water molecules from nonpolar surfaces upon complex formation.

Furthermore, the negative values of Δ*G*° at all investigated temperatures confirm that the binding of both triphenyltin(IV) compounds to **Tf** is spontaneous under physiological conditions. Taken together, these results demonstrate that the association of **Ph_3_SnL_1_** and **Ph_3_SnL_2_** with **Tf** is primarily driven by hydrophobic forces, in excellent agreement with the fluorescence quenching analysis and supporting the formation of stable ground-state protein–ligand complexes.

#### 3.1.3. Effect of Fe^3+^ Ions on the Interaction of Ph_3_SnL_1_ and Ph_3_SnL_2_ with Tf

Proteins belonging to the transferrin family are monomeric glycoproteins composed of two homologous lobes, each capable of binding one ferric ion. In biological systems, iron exists predominantly in the Fe^2+^ form in the intracellular environment, whereas Fe^3+^ is the dominant oxidation state in extracellular fluids [41,42]. **Tf** exhibits a pronounced selectivity for Fe^3+^ over Fe^2+^ and other metal ions, which arises from the specific coordination environment provided by donor atoms from amino acid side chains within its metal-binding clefts [43,44].

**Figure 5:**
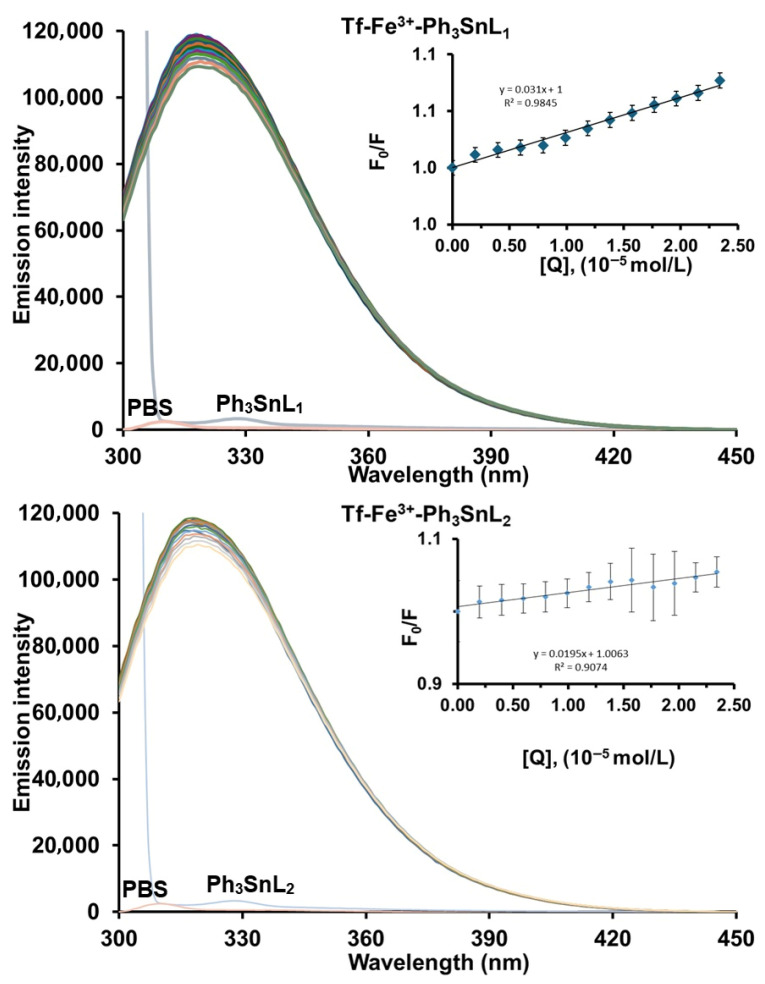
Fluorescence emission spectra of **Tf-Fe^3+^** in the presence of increasing concentrations of **Ph_3_SnL_1_** and **Ph_3_SnL_2_** (T = 296 K; pH 7.4; λex = 280 nm). [**Tf**] = [**Fe^3+^**] = 2 µM; [**Ph_3_SnL_1_] = [Ph_3_SnL_2_**] = 0–27.2 µM. The inset shows the Stern–Volmer plot.

To assess the influence of iron loading on protein–ligand interactions, the effect of Fe^3+^ ions on the binding behavior of the organotin(IV) compounds **Ph_3_SnL_1_** and **Ph_3_SnL_2_** toward **Tf** was systematically investigated [45]. A comparative analysis between the binary (**Tf–Ph_3_SnL_1_** and **Tf–Ph_3_SnL_2_**) and ternary (**Tf–Ph_3_SnL_1_–Fe^3+^** and **Tf–Ph_3_SnL_2_–Fe^3+^**) systems was performed at 296 K. The fluorescence emission spectra of the ternary systems, together with the corresponding Stern–Volmer plots, are shown in Figure 5. Similarly to the binary systems, a gradual decrease in fluorescence intensity was observed upon ligand addition, while no appreciable shift in the maximum emission occurred, indicating that the quenching mechanism remains predominantly static in nature.

Binding constants (*K_a_*) and the apparent number of binding sites (*n*) were derived from the logarithmic plots (Figure S5), and the obtained parameters are summarized in Table 3. The results clearly demonstrate that the presence of Fe^3+^ ions markedly enhances the binding affinity of both **Ph_3_SnL_1_** and **Ph_3_SnL_2_** toward **Tf**. In particular, the binding constant for **Ph_3_SnL_1_** increases from 5.72 × 10^2^ to 4.79 × 10^3^ M^−1^ upon iron coordination, while a corresponding increase from 3.93 × 10^2^ to 8.54 × 10^2^ M^−1^ is observed for **Ph_3_SnL_2_**. In parallel, an increase in the number of binding sites (*n*) is detected for both systems, suggesting improved accessibility or stabilization of ligand-binding regions in the iron-loaded protein.

The enhanced binding affinity observed in the presence of Fe^3+^ can be rationalized by iron-induced conformational changes in **Tf**. Coordination of Fe^3+^ is known to trigger a transition from the open (*apo*) to the closed (*holo*) conformation of the protein, resulting in substantial rearrangements of the binding clefts and alterations in local polarity. These structural changes likely promote stronger hydrophobic contacts and more favorable packing interactions between **Tf** and the organotin(IV) compounds, thereby facilitating more stable complex formation.

Overall, these findings highlight the crucial role of protein metallation in modulating the bio-interfacial behavior of organotin(IV) compounds. The pronounced effect of Fe^3+^ on both binding affinity and binding stoichiometry underscores the importance of considering physiologically relevant metal-loaded protein states when evaluating the biological interactions, transport, and potential biodistribution of metal-containing compounds.

### 3.2. Molecular Docking Analysis of Apo- and Holo-Transferrin Binding

The molecular docking analysis provided detailed insight into how the investigated organotin(IV) compounds interact with different domains of **Tf**, as well as how the presence of Fe^3+^ ions influences the stability of the protein–ligand compounds. The calculated binding free energies indicate that all compounds bind stably to both major domains of **Tf**, yet with clear distinctions between the *apo*- and *holo*-forms of the protein. In apotransferrin, which adopts a more open and flexible conformation, the compounds generally exhibit more favorable Δ*G*_bind_ values compared to the holoprotein. This trend is expected, as the absence of Fe^3+^ increases the mobility of residues within the binding cleft, allowing deeper ligand penetration and a broader range of van der Waals and hydrophobic contacts.

However, although the docking results favor binding to the *apo*-form, experimental findings show that the biological activity of the compounds increases in the presence of iron. This discrepancy suggests that the thermodynamics of binding predicted by docking simulations is not the sole determinant of **Tf**-mediated biological accessibility, particularly in metal-containing proteins whose functional behavior depends strongly on conformational state and the presence of the metal center. Importantly, conventional AutoDock scoring functions are not parameterized to capture metal-induced electronic polarization, coordination effects, or charge redistribution associated with Fe^3+^ binding, and therefore cannot quantitatively reflect the functional role of iron in transferrin–ligand interactions. Analysis of individual energy contributions reveals a more pronounced electrostatic component (Δ*G*_elec_) in the *holo*-form, although its absolute value remains smaller than that of the dispersion interactions. The presence of Fe^3+^ modifies the local charge distribution and rearranges the geometry of the coordination environment, which is reflected in changes in Δ*G*_vdw_ and Δ*G*_hbond_ components. In **holTf**, hydrogen bonding and specific interactions with residues that stabilize the metal center become more prominent, yet the overall binding energy remains less favorable than in **apoTf**, an expected artifact of docking methods that struggle to describe the coordination dynamics of metalloproteins accurately.

A comparison of interactions within the N- and C-lobes further revealed that the C-lobe, characterized by a more rigid architecture, provides a more stabilizing environment for the **Ph_3_SnL_2_** compound, particularly in the apo-form. In this domain, the complex exhibits more favorable internal energy contributions and reduced torsional penalties, suggesting that the C-lobe may serve as a preferential anchoring site for this ligand. Conversely, the N-lobe shows enhanced interactions for **Ph_3_SnL_1_**, facilitated by its greater conformational flexibility; however, this same flexibility increases sensitivity to structural fluctuations that are not fully captured by docking algorithms. As a result, while certain energetic terms appear more favorable in the N-lobe, the overall stability of binding remains lower than in the C-lobe.

**Figure 6:**
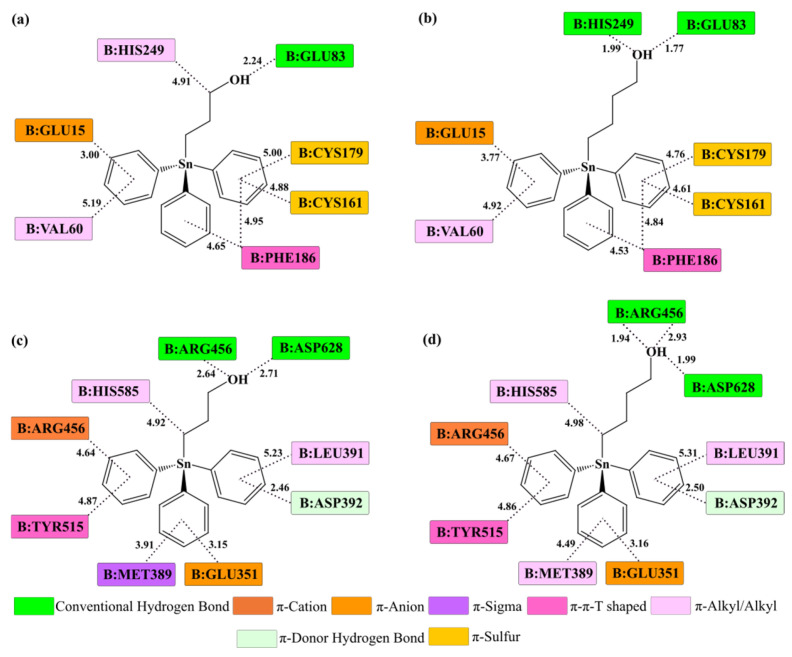
Two-dimensional representations of the key intermolecular interactions between **Ph_3_SnL_1_** (**a**,**c**) and **Ph_3_SnL_2_** (**b**,**d**) within the N-lobe (up) and C-lobe (down) binding sites of **Tf** in *apo* forms. Interatomic distances (Å) obtained from molecular docking analyses are indicated. Different colors denote distinct types of noncovalent interactions, as specified in the legend.

The molecular docking analysis provides detailed atomistic insight into the binding modes of **Ph_3_SnL_1_** and **Ph_3_SnL_2_** within the N- and C-lobe binding regions of **Tf** in both the *apo* and *holo* forms of the protein (Figure 6 and Figure S6). The obtained results indicate that binding is governed by a combination of hydrophobic contacts, π-type interactions, and hydrogen bonds, with the relative contribution of individual interaction types depending on ligand structure, lobe identity, and the degree of protein metallation.

In the apo-form of **Tf**, **Ph_3_SnL_1_** exhibits a pronounced preferential affinity toward the N-lobe. Its triphenyltin fragment is stabilized through multiple π-alkyl, π-σ, π-π, and π-sulfur interactions with amino acid residues such as Val60, Cys161, Cys179, Phe186, and His249. Additional stabilization arises from electrostatic π-cation interactions involving the side chains of polar amino acids located in the vicinity of the binding site. Moreover, the terminal hydroxyl group of the alkyl-ω-ol chain participates in hydrogen-bond formation with polar residues, such as Glu83 and His249, further contributing to complex stability. The relatively flexible architecture of the N-lobe enables efficient accommodation of the bulky organotin scaffold, favoring dispersion interactions that dominate the binding energetics within this domain.

In contrast, **Ph_3_SnL_2_** displays a stronger propensity for binding to the C-lobe, particularly in **apoTf**. As shown in Figure 6c,d, this ligand establishes an extensive network of hydrophobic interactions with residues such as Leu391, Met389, Tyr515, and His585, accompanied by additional stabilizing π-cation/π-anion interactions between the aromatic rings of the ligand and Arg456. Notably, the hydroxyl terminus of **Ph_3_SnL_2_** participates in well-defined hydrogen bonds with Asp628 and Arg456, contributing to improved geometric complementarity, more effective ligand anchoring, and reduced torsional strain. The more rigid structural framework of the C-lobe clearly favors this ligand, providing a more stable and better-defined binding environment compared to the more flexible N-lobe.

Upon coordination of Fe^3+^ ions, no fundamental changes in the dominant types of intermolecular interactions are observed for either ligand; however, subtle variations in interaction geometry and interatomic distances do occur (Figure S6). In **holoTf**, closure of the metal-binding clefts leads to reorganization of the local coordination environment and changes in intramolecular distances within the binding sites. For both **Ph_3_SnL_1_** and **Ph_3_SnL_2_**, interactions with amino acid residues involved in stabilization of the Fe^3+^ coordination center become more pronounced, particularly through hydrogen bonding and polar contacts. Nevertheless, hydrophobic and dispersion interactions remain the dominant contributors to overall binding stability, in full agreement with the thermodynamic signatures derived from fluorescence experimental measurements.

### 3.3. ONIOM (QM/MM) Analysis of Transferrin–Ligand Interactions

ONIOM (QM/MM) interaction energies were calculated for the lowest-energy docking poses to refine the description of transferrin–ligand binding beyond the empirical scoring function. As summarized in Table 4, all complexes exhibit favorable interaction energies (Δ*E*_int_ = −38 to −73 kcal mol^−1^), confirming that both **Ph_3_SnL_1_** and **Ph_3_SnL_2_** are efficiently stabilized within transferrin binding pockets when electronic effects at the bio-interface are explicitly considered. Although the ONIOM QM/MM interaction energies Δ*E*_int_ are strongly stabilizing, they should not be directly equated with the experimental binding constants (*K*_a_). The experimentally derived *K*_a_ reflects the solution-phase binding equilibrium and is related to the Gibbs free energy (Δ*G*°), which includes solvation and entropic contributions as well as ensemble averaging over multiple binding microstates, whereas Δ*E*_int_ describes the stabilization of a selected bound microstate within a cluster-based ONIOM framework and is largely enthalpy-like. Consequently, strong local interfacial stabilization can coexist with moderate macroscopic affinities, consistent with the positive Δ*H*° and Δ*S*° values and a predominantly hydrophobically driven binding mechanism.

A comparison with docking-derived Δ*G*_bind_ values reveals a consistent trend for the apo-form, where more favorable docking scores are accompanied by strongly stabilizing ONIOM interaction energies. In **apoTf**, the most favorable case (C-lobe–**Ph_3_SnL_2_**) shows both the best docking affinity (Δ*G*_bind_ = −10.27 kcal mol^−1^) and the strongest ONIOM stabilization (Δ*E*_int_ = −73 kcal mol^−1^), indicating that the rigid C-lobe architecture enables optimal dispersion-dominated packing of the bulky organotin scaffold. By contrast, weaker stabilization is observed for C-lobe– **Ph_3_SnL_1_** (Δ*E*_int_ = −38 kcal mol^−1^), supporting the docking-based preference of **Ph_3_SnL_1_** toward the N-lobe.

Importantly, docking predicts slightly weaker binding for **holoTf**, whereas fluorescence experiments demonstrate enhanced affinity in the presence of Fe^3+^. ONIOM results help to rationalize this apparent discrepancy. Although the *holo* complexes show somewhat less favorable docking Δ*G*_bind_ values, their QM/MM interaction energies remain strongly stabilizing (Δ*E*_int_ ≈ –60 to –66 kcal mol^−1^), indicating that binding remains energetically accessible after iron-induced closure of the lobes. This behavior is consistent with the experimental thermodynamic signature (positive Δ*H*° and Δ*S*°), which indicates that the association is primarily driven by hydrophobic/dispersion interactions and by metal-induced modulation of pocket accessibility, rather than by the absolute docking score. In other words, Fe^3+^ coordination can enhance functional binding by reshaping the microenvironment and facilitating productive packing and presentation of the ligand, even if the rigidified *holo* pocket is penalized by docking scoring terms.

## 4. Conclusions

This work delivers a coherent spectroscopic and multiscale computational dissection of the bio-interfacial behavior of two tunable (alkyl-ω-ol)triphenyltin(IV) compounds, **Ph_3_SnL_1_** and **Ph_3_SnL_2_**, toward human serum transferrin (**Tf**). Steady-state fluorescence measurements demonstrated efficient quenching of **Tf** emission without meaningful spectral shifts, indicating formation of stable, non-fluorescent ground-state complexes. The apparent quenching rate constants (kq ~10^12^ M^−1^ s^−1^), exceeding the diffusion-controlled limit by orders of magnitude, strongly support that collisional quenching is not the principal mechanism and that static complexation dominates the fluorescence response. Binding analysis revealed moderate affinity and a near-unimodal binding stoichiometry (n ≈ 0.65–0.90), with negative Gibbs free energies (Δ*G*° < 0) supporting spontaneity across the investigated temperature range. The uniformly positive van’t Hoff signatures (Δ*H*° > 0; Δ*S*° > 0) indicate that the association process is entropically favored and largely driven by hydrophobic and dispersion-type contributions.

The presence of Fe^3+^ markedly strengthened ligand engagement with **Tf,** particularly for **Ph_3_SnL_1_**, evidencing that iron-induced structural closure of the protein lobes reshapes the polarity and accessibility of binding pockets relevant for organotin scaffold recognition. Molecular docking validated lobe-selective binding microprofiles: **Ph_3_SnL_2_** forms more persistent, geometrically well-packed complexes within the rigid C-lobe, while **Ph_3_SnL_1_** preferentially penetrates the more adaptive N-lobe. In both lobes, complex stability is predominantly secured by hydrophobic contacts and π-type interactions, complemented by a secondary network of hydrogen bonds and polar contacts. ONIOM QM/MM refinements of the most stable docking poses yielded strongly favorable interaction energies (Δ*E*_int_ ≈ –38 to –73 kcal mol^−1^), supporting the energetic feasibility of binding even after iron-mediated lobe rigidification and offering a more realistic description of interfacial charge–packing interplay than empirical docking scoring alone.

The conclusions of this study are based on a targeted model system comprising two closely related triphenyltin(IV) ligands investigated under in vitro and in silico conditions. While this approach enables a controlled dissection of structure–interaction relationships at the transferrin interface, it does not capture potential competition with endogenous ligands, the influence of systemic iron homeostasis, or cellular uptake and trafficking processes. Addressing these aspects will require complementary cellular and in vivo studies and represents an important direction for future work.

Collectively, the results define how subtle ligand architectural differences, lobe topology, and protein metallation jointly modulate the stability and microprofile of organotin(IV) complexes at the transferrin bio-interface. The study furnishes a reproducible molecular-level foundation for predicting and steering the interaction behavior of structurally related organometallic systems in serum-rich environments.

## Figures and Tables

**Table 1:** The Stern–Volmer quenching parameters (*K*_SV_) and bimolecular quenching rate constants (*K*_q_) for the interaction of transferrin with **Ph_3_SnL_1_** and **Ph_3_SnL_2_** at different temperatures.

System	T (K)	*K*_SV_ × 10^3^ (M^−1^)	*K_q_* (M^−1^)	R^2^ *^a^*
**Tf-Ph_3_SnL_1_**	296	2.24 ± 0.03	2.24 × 10^11^	0.902
	303	5.21 ± 0.03	5.21 × 10^11^	0.945
	310	5.30 ± 0.03	5.30 × 10^11^	0.961
**Tf-Ph_3_SnL_2_**	296	1.02 ± 0.01	1.02 × 10^11^	1.000
	303	4.95 ± 0.03	4.95 × 10^11^	0.899
	310	5.45 ± 0.03	5.45 × 10^11^	0.952

*^a^* R is the correlation coefficient for Stern–Volmer plots.

**Table 2:** Binding parameters and thermodynamic signatures for the interaction of **Tf** with **Ph_3_SnL_1_** and **Ph_3_SnL_2_** at different temperatures.

System	*T*(K)	*K*_a_ (M^−1^)	*n*	R^2^ *^a^*	Δ*H*^0^ (KJmol^−1^)	Δ*S*^0^ (JK^−1^mol^−1^)	Δ*G*^0^ (KJmol^−1^)
**Tf-Ph_3_SnL_1_**	296	(5.72 ± 0.03) × 10^2^	0.85	0.953	65.170	0.273	−15.68
	303	(1.12 ± 0.02) × 10^3^	0.87	0.965			−16.23
	310	(1.89 ± 0.03) × 10^3^	0.90	0.961			−19.50
**Tf-Ph_3_SnL_2_**	296	(3.93 ± 0.03) × 10^2^	0.65	0.999	88.586	0.348	−14.59
	303	(7.77 ± 0.02) × 10^2^	0.73	0.996			−15.28
	310	(2.00 ± 0.01) × 10^3^	0.82	0.979			−19.46

*^a^* R is the correlation coefficient.

**Table 3:** Interaction parameters for binary (**Tf–Ph_3_SnL_1_** and **Tf–Ph_3_SnL_2_**) and ternary (**Tf–Ph_3_SnL_1_–Fe^3+^** and **Tf–Ph_3_SnL_2_–Fe^3+^**; **Tf**:Fe^3+^ = 1:1) systems at 296 K.

System	*K*_sv_ × 10^3^ (M^−1^)	R^2^	*K*_a_ (M^−1^)	*n*	R^2^ *^a^*
**Tf-Ph_3_SnL_1_**	2.24 ± 0.028	0.943	(5.72 ± 0.05) ×10^2^	0.85	0.953
**Tf-Ph_3_SnL_1_-Fe^3+^**	3.10 ± 0.021	0.985	(4.79 ± 0.03) × 10^3^	1.10	0.903
**Tf-Ph_3_SnL_2_**	1.02 ± 0.011	0.971	(3.93 ± 0.03) ×10^2^	0.65	0.999
**Tf-Ph_3_SnL_2_-Fe^3+^**	1.95 ± 0.028	0.907	(8.54 ± 0.02) × 10^2^	0.72	0.997

*^a^* R is the correlation coefficient.

**Table 4:** Thermodynamic and QM/MM (ONIOM) interaction parameters (kcal mol^−1^) for the most stable conformation of the investigated compound bound to different active sites of human serum transferrin (**apoTf** and **holoTf**), as determined by molecular docking and ONIOM QM–MM energy analysis: inhibition constants (*K*_i_, µM), binding free energy (Δ*G*_bind_), torsional energy (Δ*G*_tor_), electrostatic contribution (Δ*G*_elec_), van der Waals component (Δ*G_vdw+hbond+desolv_*), hydrogen bonding (Δ*G*_hbond_), desolvation effects (Δ*G*_desolv_), and ONIOM QM/MM interaction energy (Δ*E*_int_).

Complex	ΔG*_bind_*	Δ*E_i_*_nt_	K_i_ (nM)	ΔG*_inter_*	ΔG*_vdw+hbond+desolv_*	ΔG*_elec_*	ΔG*_total_*	ΔG*_tor_*	ΔG*_unb_*
Apotransferrin (**apoTf**)									
**N-Lobe-** **Ph_3_SnL_1_**	−9.25	−67	164.5	−11.17	−11.08	−0.09	−1.55	1.92	−1.55
**C-Lobe-** **Ph_3_SnL_1_**	−9.12	−38	207.9	−11.04	−10.91	−0.13	−1.43	1.92	−1.43
**N-Lobe-** **Ph_3_SnL_2_**	−9.67	−65	81.9	−11.86	−11.59	−0.28	−1.67	2.20	−1.67
**C-Lobe-** **Ph_3_SnL_2_**	−10.27	−73	29.77	−12.46	−12.15	−0.31	−1.64	2.20	−1.64
Holotransferrin (**hTf**)									
**N-Lobe-** **Ph_3_SnL_1_**	−9.03	−60	239.8	−10.95	−10.84	−0.11	−1.56	1.92	−1.56
**C-Lobe-** **Ph_3_SnL_1_**	−8.97	−62	267.4	−10.89	−10.72	−0.17	−1.57	1.92	−1.57
**N-Lobe-** **Ph_3_SnL_2_**	−9.53	−60	103.0	−11.73	−11.51	−0.22	−1.55	2.20	−1.55
**C-Lobe-** **Ph_3_SnL_2_**	−10.02	−66	45.49	−12.21	−11.89	−0.32	−1.62	2.20	−1.62

## Data Availability

The original contributions presented in this study are included in the article/supplementary material. Further inquiries can be directed to the corresponding authors.

## Supplementary Materials

The following supporting information can be downloaded at https://www.mdpi.com/article/10.3390/ma19030457/s1., Figure S1. Emission spectra of **Tf** in the presence of increasing concentrations of **Ph_3_SnL_1_** (T = 303 K; pH 7.4; λ_ex_ = 280 nm). [**Tf**] = 2 µM; [**Ph_3_SnL_1_] =** 0–27.2 µM. The inset shows the Stern–Volmer plot.; Figure S2. Emission spectra of **Tf** in the presence of increasing concentrations of **Ph_3_SnL_1_** (T = 310 K; pH 7.4; λ_ex_ = 280 nm). [**Tf**] = 2 µM; [**Ph_3_SnL_1_] =** 0–27.2 µM. The inset shows the Stern–Volmer plot.; Figure S3. Emission spectra of **Tf** in the presence of increasing concentrations of **Ph_3_SnL_2_** (T = 303 K; pH 7.4; λ_ex_ = 280 nm). [**Tf**] = 2 µM; **[Ph_3_SnL_2_**] = 0–27.2 µM. The inset shows the Stern–Volmer plot.; Figure S4. Emission spectra of **Tf** in the presence of increasing concentrations of **Ph_3_SnL_2_** (T = 310 K; pH 7.4; λ_ex_ = 280 nm). [**Tf**] = 2 µM; **[Ph_3_SnL_2_**] **=** 0–27.2 µM. The inset shows the Stern–Volmer plot.; Figure S5. The plots of log (*F*_0_ − *F*)/*F* vs. log [Q] at 296 K.; Figure S6. Two-dimensional representations of the key intermolecular interactions between **Ph_3_SnL_1_** (**a**,**c**) and **Ph_3_SnL_2_** (**b**,**d**) within the N-lobe (up) and C-lobe (down) binding sites of **Tf** in *holo* forms. Interatomic distances (Å) obtained from molecular docking analyses are indicated. Different colors denote distinct types of noncovalent interactions, as specified in the legend.
