# Supplementary material for: Unveiling the Bio-Interface via Spectroscopic and Computational Studies of (Propyl-3-ol/butyl-4-ol)triphenyltin(IV) Compound Binding to Human Serum Transferrin
Source: Materials (Basel). 2026 Jan 23;19(3):457. doi: 10.3390/ma19030457 (PMC12898580; doi:10.3390/ma19030457)
Supplement: Supplementary file 1 [file materials-19-00457-s001.zip › materials-4100436-supplementary.pdf]

Supporting Information

Unveiling the Bio-Interface via Spectroscopic and Computational Studies of (Propyl-3-ol/butyl-4-ol)triphenyltin(IV) Compounds Binding to Human Serum Transferrin

Žiko Milanović [1,*], Emina Mrkalić [1], Jovan Kulić [2], Goran N. Kaluđerović [3,*]

[1] *Institute for Information Technologies, University of Kragujevac, Liceja Kneževine Srbije 1A, 34000 Kragujevac, Serbia*

[2] *Faculty of Medicine Foča, University of East Sarajevo, Foča, Republic of Srpska, Bosnia and Herzegovina*

[3] *Department of Engineering and Natural Sciences, University of Applied Sciences Merseburg, Eberhard-Leibnitz-Straße 2, 06217 Merseburg, Germany*

* *Correspondence: ziko.milanovic@uni.kg.ac.rs (Ž.M.); goran.kaluderovic@hs-merseburg.de (G.N.K.)*

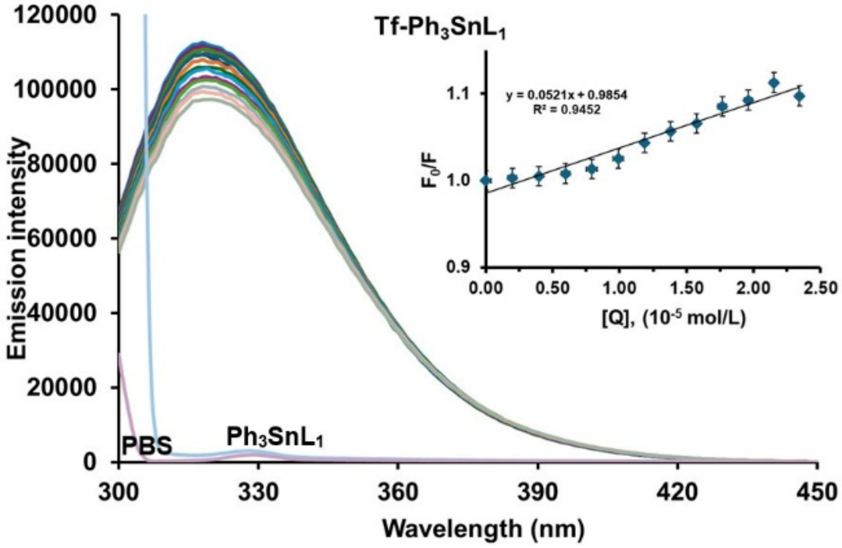

Figure S1. Emission spectra of **Tf** in the presence of increasing concentrations of **Ph_3SnL_1** (T = 303 K; pH 7.4; λ_{ex} = 280 nm). [**Tf**] = 2 μM; [**Ph_3SnL_1**] = 0 – 27.2 μM. The inset shows the Stern-Volmer plot.

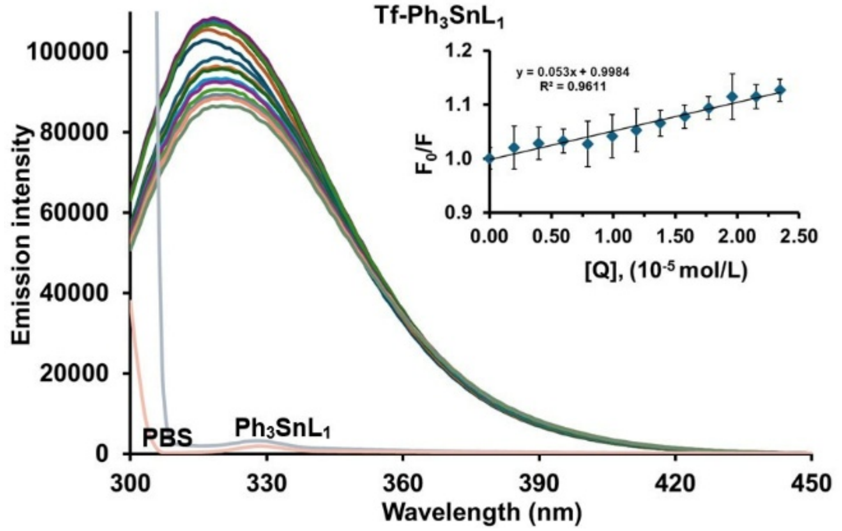

Figure S2. Emission spectra of **Tf** in the presence of increasing concentrations of **Ph_3SnL_1** (T = 310 K; pH 7.4; λ_{ex} = 280 nm). [**Tf**] = 2 μM; [**Ph_3SnL_1**] = 0 – 27.2 μM. The inset shows the Stern-Volmer plot.

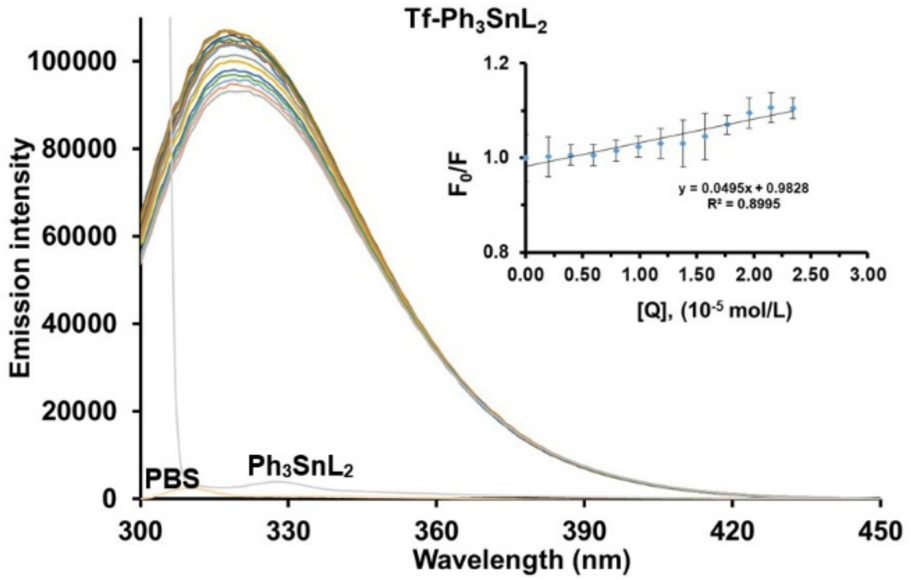

Figure S3. Emission spectra of **Tf** in the presence of increasing concentrations of **Ph_3SnL_2** (T = 303 K; pH 7.4; λ_{ex} = 280 nm). [**Tf**] = 2 μM; **[Ph_3SnL_2]** = 0 – 27.2 μM. The inset shows the Stern-Volmer plot.

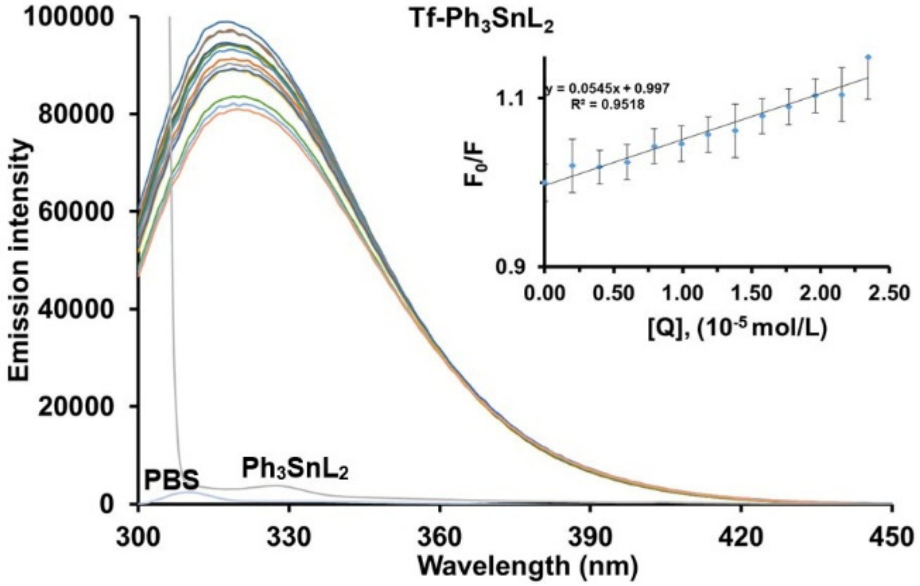

Figure S4. Emission spectra of **Tf** in the presence of increasing concentrations of **Ph_3SnL_2** (T = 310 K; pH 7.4; λ_{ex} = 280 nm). [**Tf**] = 2 μM; **[Ph_3SnL_2] =** 0 – 27.2 μM. The inset shows the Stern-Volmer plot.

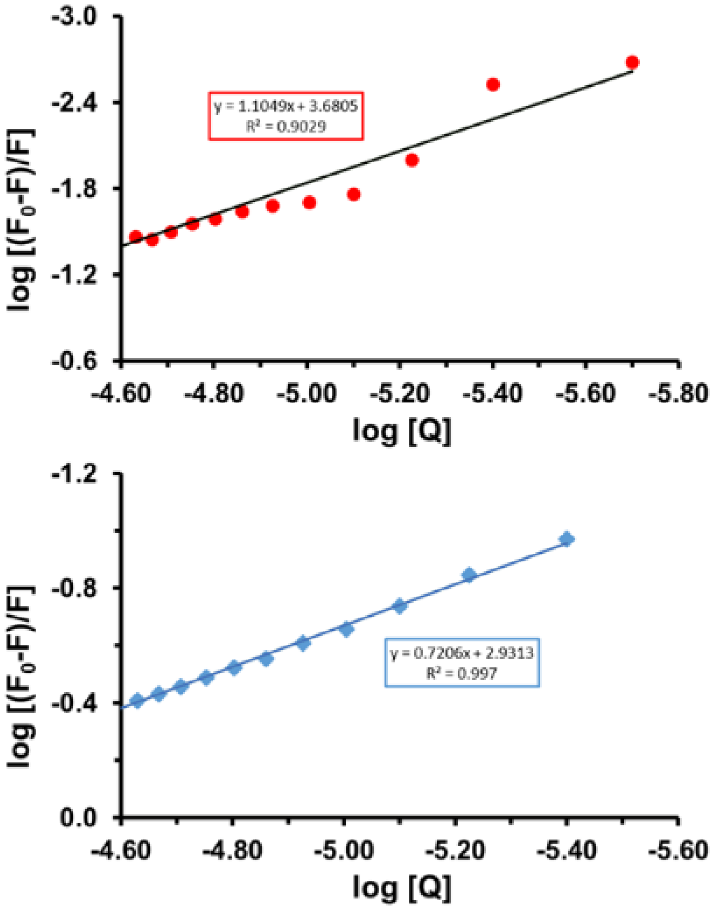

Figure S5. The plots of log ($F_0 - F$)/F vs. log [Q] at 296 K for **Tf-Ph$_3$SnL$_1$** (up) and **Tf-Ph$_3$SnL$_2$** (down).

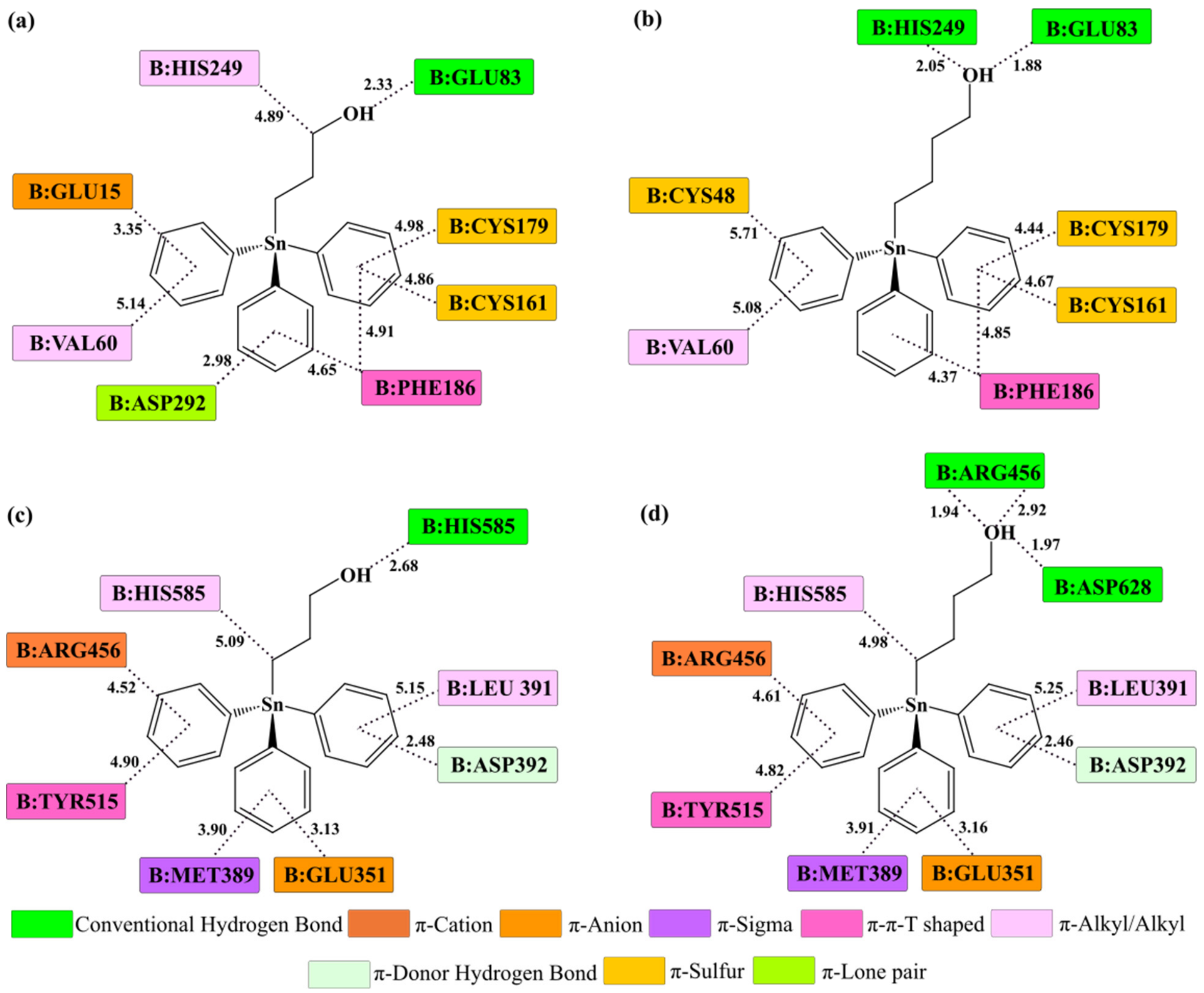

Figure S6. 2D representations of the key intermolecular interactions between **Ph_3SnL_1** (a, c) and **Ph_3SnL_2** (b, d) within the N-lobe (up) and C-lobe (down) binding sites of **Tf** in holo forms. Interatomic distances (Å) obtained from molecular docking analyses are indicated. Different colors denote distinct types of noncovalent interactions, as specified in the legend.
